# Recent Advances in the Application of *Selectfluor^TM^F-TEDA-BF_4_* as a Versatile Mediator or Catalyst in Organic Synthesis 

**DOI:** 10.3390/molecules16086432

**Published:** 2011-07-29

**Authors:** Stojan Stavber

**Affiliations:** 1 Laboratory for Organic and Bioorganic Chemistry, “Jožef Stefan” Institute, Jamova 39, 1000 Ljubljana, Slovenia; 2 Centre of Excellence CIPKeBiP, Jamova 39, 1000 Ljubljana, Slovenia; Email: stojan.stavber@ijs.si; Tel.: +386-1-477-3660, Fax: +386-1-423-5400

**Keywords:** Selectfluor^TM^ F-TEDA-BF_4_, oxidative transformations, coupling reactions, halogenation

## Abstract

*Selectfluor^TM^* F-TEDA-BF_4_ (1-chloromethyl-4-fluoro-1,4-diazoniabicyclo [2.2.2]octane bis(tetrafluoroborate) is not only one of the most efficient and popular reagents for electrophilic fluorination, but as a strong oxidant is also a convenient mediator or catalyst of several “fluorine-free” functionalizations of organic compounds. Its applications as a mediator in transformations of oxidizable functional groups or gold-catalyzed C-C and C-heteroatom oxidative coupling reactions, a catalyst in formation of various heterocyclic rings, a reagent or catalyst of various functionalizations of electron-rich organic compounds (iodination, bromination, chlorination, nitration, thiocyanation, sulfenylation, alkylation, alkoxylation), a catalyst of one-pot-multi-component coupling reactions, a catalyst of regioselective ring opening of epoxides, a deprotection reagent for various protecting groups, and a mediator for stereoselective rearrangement processes of bicyclic compounds are reviewed and discussed.

## 1. Introduction

**Figure 1:**
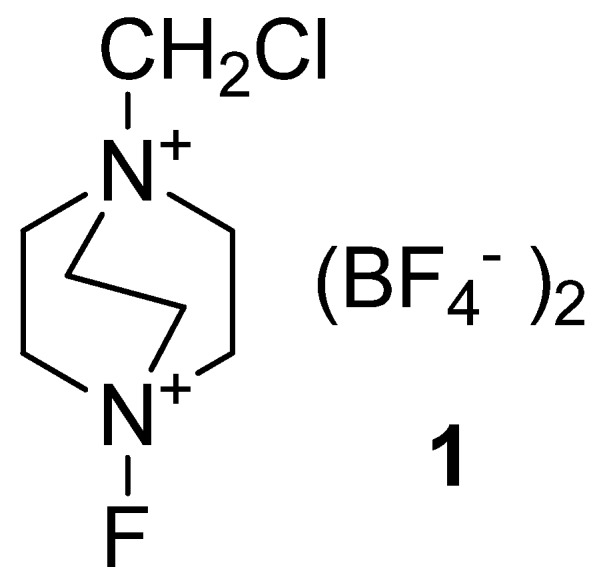
1-Chloromethyl-4-fluoro-1,4-diazoniabyciclo[2.2.2]octane bis(tetrafluoroborate) *Selectfluor F-TEDA-BF_4_* .

Selective fluorofunctionalisation of organic compounds under mild reaction conditions following an electrophilic reaction process is one of the most important strategic approaches in the organic synthesis of fluoro-substituted organic derivatives, chemicals of wide interest to the basic and applied research community [1,2,3]. The group of agents enabling this type of functionalisation are known as “electrophilic fluorinating reagents”, and besides molecular fluorine, include three main groups of reagents; xenon fluorides, fluoroxy compounds and N-F compounds. Organic compounds bearing a reactive N-F bond were introduced as mild reagents for selective introduction of a fluorine atom into organic compounds less than 25 years ago by the efforts of Umemoto`s group, leading to the first isolatable N-fluoropyridinium salts, their application for fluorofunctionalization of organic compounds, and soon after, also to their commercial production [4,5]. These easily-handled “bench-top” chemicals, usually with optimal stability/reactivity characteristics, have practically revolutionized the common perception of synthesis of site-selective fluoro-substituted organic compounds, and brought this important task in organic synthesis to the status of an ordinary experimental procedure suitable for any organic chemistry laboratory [3,6,7,8]. The main N-fluoro reagents families are neutral N-fluoro amines or amides, N-fluoropyridinium salts and quaternary N-fluoro salts, and the most often used members of the last group are the N-fluoro derivatives of 1,4-diazoniabicyclo[2.2.2]octane (triethylendiamine; TEDA), among which 1-chloromethyl-4-fluoro-1,4-diazoniabyciclo[2.2.2]octane bis(tetrafluoroborate) (**1**, Figure 1) known under the trade name of *Selectfluor^TM^ F-TEDA-BF_4_* is the most representative and widely used in this series.

Since its discovery [9] and academic introduction [10] twenty years ago, *Selectfluor^TM^ F-TEDA-BF_4_* quickly became one of the most popular reagents for electrophilic fluorination of organic compounds [11,12,13,14], not only as an ordinary reagent at the laboratory level, but also as multi-ton scale material produced for several industrial applications [15]. Its thermal stability (up to 195 °C), moderate to high solubility and stability in polar solvents (water, acetonitrile, DMF, methanol, nitromethane, THF) [16], and low toxicity [13,15] are characteristics giving F-TEDA-BF_4_ its utility, while its half-wave potential against SCE as high as 0.33 V [17] makes it one of the most powerful oxidants in the N-F compounds series [18] and therefore a convenient moderator of many “other-than-fluorine” functionalizations of organic compounds. The literature data dealing with *Selectfluor^TM^ F-TEDA-BF_4_* as a fluorinating reagent have been comprehensively surveyed during last 15 years [1,6,7,8,11,12,13,14,8,11], while its role in other transformations has been reviewed separately [19]; newer literature and recent advances on this topics are thesubject of the present account.

## 2. Functionalizations of Organic Compounds with *Selectfluor* F-TEDA-BF_4_ Other than Fluorine

### 2.1. Transformations of Oxidizable Functional Groups

**Scheme 1:**
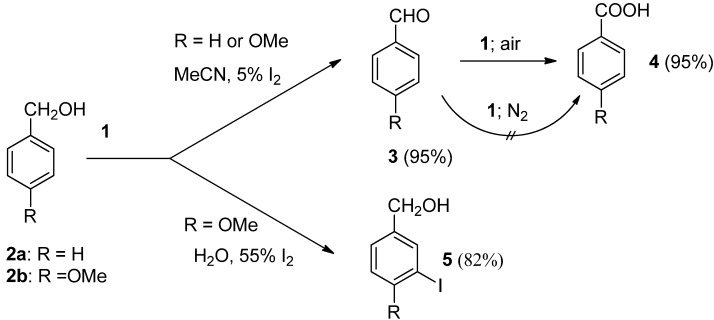
Reactions of benzylic alcohols with *Selectfluor F-TEDA-BF_2_* **1**.

In the presence of chemicals having oxidizing power the hydroxyl functional group could often be transformed to various kinds of carbonyl functionalitiy. Primary benzylic alcohols were found to be relatively stable towards **1** since their transformations with **1** in acetonitrile media to moderate amounts of corresponding aldehydes, and further to benzoic acid derivatives, needs long reaction times (15-435 hours) and reflux temperature. It was also established that aromatic aldehydes could be transformed with **1** to benzamides or benzoates after reaction in the presence of amines or alcohols, but again the long reaction time (40-70 hours) required for these functionalisations makes them less attractive [20]. On the other hand, catalytic amounts of molecular iodine enhance the reaction and its efficiency considerably. Benzyl alcohol (**2a**) and 4-methoxybenzyl alcohol (**2b**) were thus readily transformed to their aldehydes and further to benzoic acid derivatives (**3** and **4**) after 2 hours treatment with **1** in MeCN solution in the presence of 5 mol% of I_2_ under an air atmosphere (Scheme 1), while in the case of the treatment of **2b** in aqueous media and in the presence of 55 mol% of iodine, the benzylic hydroxyl group remained unattached and iodo-functionalization of the aromatic ring to **5** took place [21]. Alkyl alcohols could also be readily transformed by **1** to their carbonyl derivatives [22].

**Scheme 2:**
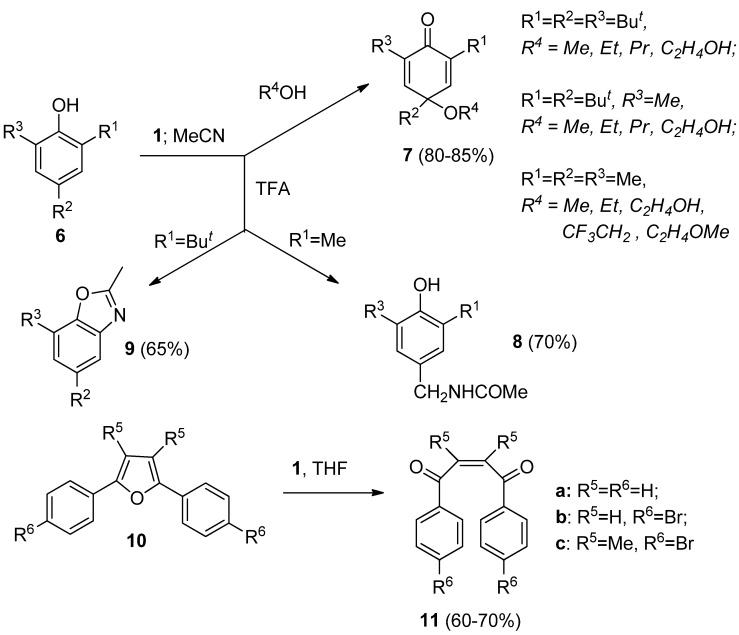
Transformations of 2,4,6-trialkyl substituted phenols and 2,5-diarylfurans with *Selectfluor F-TEDA-BF_2_* **1**.

Reactions of phenols with **1** were intensively studied. Phenols substituted by an additional hydroxy substituent at the *ortho* or *para* position were readily oxidized to the corresponding quinones when treated by **1** in MeCN [22], while the course of reaction of 2,4,6-trialkyl substituted phenols with **1** was found to be strongly dependent on the structure of the target compounds **6** and the reaction media used (Scheme 2). Reactions in pure MeCN gave fluorinated products, while in the presence of alcohols or water *para*-quinols or *para*-quinol ethers **7** were formed in moderate to high yield. The presence of a more acidic nucleophile, such as trifluoroacetic acid (TFA), caused quite different transformations and Ritter-type functionalisation at the 4-benzylic position resulted in the formation of 4-methylacetamido-2,6-dialky substituted phenol derivatives **8**, while after *ipso* attack at position 2, followed by dealkylation and internal cyclisation, alkyl substituted benzoxazole derivatives **9** were formed [23,24]. Another oxidative transformation of oxygen containing functional moieties with **1** was found to be the ring opening of 2,5-diaryl substituted furans **10**, resulting in the stereoselective formation of *cis*-1,2-dibenzoyldione derivatives **11** [25].

**Scheme 3:**
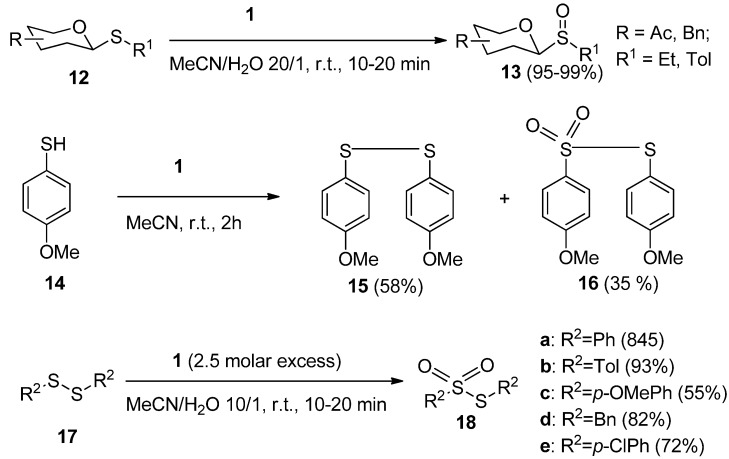
Transformations of sulfur-containing functional groups with *Selectfluor F-TEDA-BF_2_* **1**.

Sulfur-containing functional groups are usually very sensitive to oxidation. The mild oxidative nature of **1** was efficiently used advantageously in glycoside chemistry in the case of the development of a selective and efficient method for the oxidation of thioglycosides to their corresponding sulfoxide derivatives. A variety of thioglycosides (**12**, Scheme 3) were thus readily transformed to their sulfinyl derivatives **13** by treatment with a moderate molar excess of **1** in aqueous MeCN (MeCN/H_2_0 = 20/1) at room temperature for a few minutes [26]. The thiophenolic functionality was found to be more unstable towards **1** than its phenolic analogues and could be readily transformed to disulfides and further to sulfonates [22], and this path was accepted as a methodology for concise synthesis of thiosulfonates. Symmetric aromatic or benzylic disulfides **17** were thus efficiently transformed to thiosulfonates **18** with a 2.5 fold molar excess of **1** in aqueous MeCN [27], while alkyl phenyl sulfides under these reaction conditions with an equimolar amounts of **1** gave selectively the sulfoxide functionality [28]. 

An amino functional group bonded to an aromatic ring usually cannot survive the presence of **1** and demands protection by acetylation, while primary, secondary or tertiary aliphatic amines can be transformed by **1** to N-fluoro-substituted derivatives, often selectively and in moderate to good yield [14]. On the other hand, amides are relatively stable towards oxidation to imides, and up to now only a few efficient methods for direct preparation of these valuable chemicals are known, but recently the combination of the copper(I) moiety and Selecfluor F-TEDA-BF_4_ was introduced as an efficient and selective reagent system for the oxidation of amides to imides [29]. A variety of amides **19** were thus efficiently transformed to their imide derivatives **20** using the combination of **1** (2.5 equiv)/CuBr (1.2 equiv) in MeCN at room temperature (Table 1). 

**Table 1:** Oxidation of amides **19** to imides **20** using *Selectfluor F-TEDA-BF_4_*/CuBr tandem.^ a^ 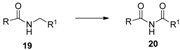

Entry	R	R ^1^	Yield (%)
1	Ph	CH_2_CH(Me)_2_	88
2	Ph	Et	77
3	Ph	C_2_H_4_COOMe	82
4	Ph	Ph	84
5	Ph	(CH_2_)_5_OCOPh	84
6	4-F-Ph	Et	80
7	4-F-Ph	*c*-C_6_H_11_	50
8	Me	Ph	83
9	*n-*C_6_H_13_	CH_2_CH(Me)_2_	79

^a^ Reaction conditions: amide **19** (0.25 mmol), *Selectfluor F-TEDA-BF_4_* (0.625 mmol), CuBr (0.3 mmol added in six portions over 40 min), MeCN (5 mL), r.t., 1 hour.

**Scheme 4:**
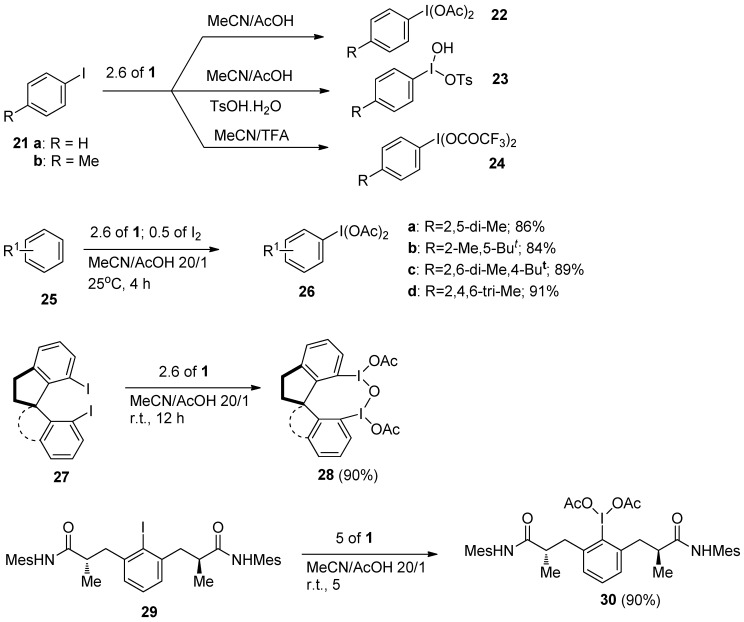
Synthesis of hypervalent iodine(III) compounds using *Selectfluor F-TEDA-BF_4_*.

Hypervalent iodine(III) compounds are valuable and versatile reagents in organic synthesis. It has been demonstrated that various types of aryl hypervalent iodine(III) compounds could be efficiently prepared using *Selectfluor F-TED-BF_4_* starting from the corresponding aryl iodides (**21**, Scheme 4), or even straightforwardly from arenes **25** following **1** mediated oxidative iodination and further in situ functionalization of aryl iodides. Using one or other approach, a variety of phenyliodine(III)diacetates **22**, **26** or phenyliodine(III)ditrifluoromethylacetates **24** were prepared with a 2.6 fold molar excess of **1** in MeCN solution in the presence of acetic or trifluoromethyl acetic acid, while in the presence of TsOH.H_2_O, Koser`s reagents **23** were synthesized [30]. The same methodology was applied for the synthesis of chiral hypervalent iodine(III) reagents **28** [31] and **30** [32], and further used for various enantioslective transformations. 

### 2.2. Oxidative Halogenation

Halogenation of organic compounds using the oxidative approach mediated by Selectfluor F-TEDA-BF_4_ has been introduced in our laboratory [33] and the methodology originally applied for the regioselective iodination of aromatic ethers using molecular iodine. Regioselective iodination at the *para* position took place, while when this position was occupied, regioselective *ortho* iodofunctionalization took place. Acetonitrile was found to be the best medium for these transformations and 50 mol% of molecular iodine was found to be enough for complete transformation of starting the material. This methodology has been intensively used for efficient and selective iodination of alkyl-substituted benzene derivatives [34], also those sterically hindered [35], as well for iodofunctinalization of arenes in ionic liquids as the reaction media [36]. 

**Scheme 5:**
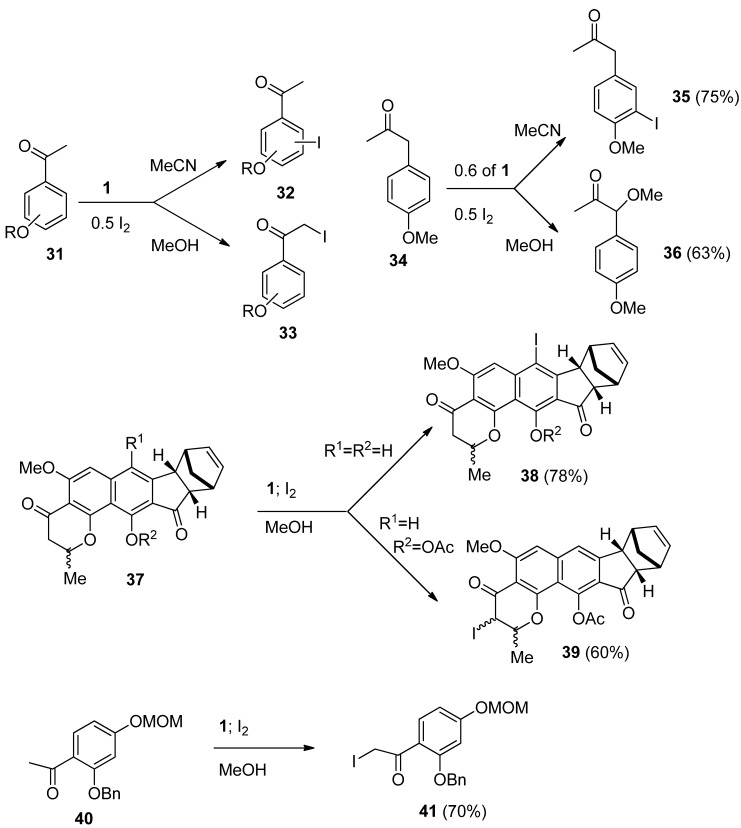
Oxidative iodination of organic compounds mediated by *Selectfluor F-TEDA-BF_4_*. The original idea and recent applications.

We have also demonstrated that the regioselectivity of iodination could be regulated by the solvent used. In the case of iodination of substituted aryl-alkyl ketones regioselective functionalization of the aromatic ring took place (**32**, Scheme 5) when the reactions were performed in MeCN, while regioselective iodination of the side chain (*eg*. **33**) has been found in reactions performed in MeOH [37]. It has been established that the stoichiometry of the process for substrate/I_2_/F-TEDA-BF_4_ is 1/0.5/0.6. This MeOH directed and F-TEDA-BF_4_ mediated iodination methodology was applied for side chain iodination of a variety of acetyl substituted aromatic compounds [38], and indanone and tetralone derivatives [39] bearing a strongly activated aromatic ring; these achievements have been reviewed in our previous account [19]. 1-(4-Methoxyphenyl)propan-2-one (**34**) was further chosen as a model substrate; in MeCN ring iodination forming **35** was established, in MeOH exclusive side-chain methoxy functionalization at the benzylic position took place (**36**), while in water regioselectivity was lost and a mixture of ring and side-chain functionalized products were observed in the crude reaction mixture [21]. Recently application of the method was successfully demonstrated for the synthesis of euplectin, where by varying the substituents on the euplectin precursor **37**, the regioselectivity of the F-TEDA-BF_4_ mediated iodination could be directed towards aryl ring iodofuctionalization resulting in **38**, or to the α-to carbonyl position resulting in **39** [40], and for side chain iodination of the protected 2,4-dihydroxy acetophenone derivative **40** to **41**, one of precursors in total synthesis of glyceollin I [41].

*Selectfluor F-TEDA-BF_4_* mediated iodination of dimethoxybenzenes (**42**, Table 2) was studied and the role of reaction media and the relative ratio of reactants on the course of the transformation evaluated. In the case of 1,2- (**42a**) and 1,4-dimethoxybenzene (**42c**) equimolar amounts of all three reactants (B) were found to be necessary for high conversion of starting material (entries 1-3 and 8,9 in Table 2), while for the iodofunctionalization of 1,3-dimethoxybenzene **42b** to **43b** a 0.5 molar amount of iodine and 0.6 molar amount of F-TEDA-BF_4_ (A) was enough for high yield iodination in all three solvents (entries 4-6). This result was explained by the different nature of the reaction path and a predominantly ionic process was proposed for case A, where iodine has the role of activator of the system and F-TEDA-BF_4_ the role of activator and regenerator of iodide liberated during the iodination process, while in the case of B, a reaction course through single electron transfer was proposed [21].

Bromination and chlorination of various unsaturated organic compounds mediated by F-TEDA-BF_4_ have also been demonstrated. Electrophilic bromination or chlorination of benzene derivatives was reported at room temperature using the anionic precursors of bromide or chloride transformed *in situ* into their electrophilic species by **1** [42]. Acetonitrile was found to be the best choice for the reaction medium, while reactions did not proceed in MeOH. A number of olefins were oxidative brominated using the F-TEDA-BF_4_/KBr tandem and for different types of substrates, addition, monobromine-substituted, or Hunsdiecker-Borodin reaction products were readily obtained [43].

**Table 2:** Iodination of dimethoxy benzenes with elemental iodine mediated by F-TEDA-BF_4_. 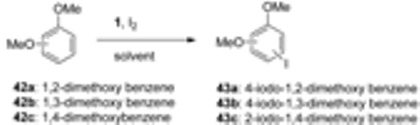

Entry	**Substrate**	Solvent	T/t (°C/h)	Reactants ratio ^a^	**Product**	Yield (%) ^b^
1		MeCN	20/4	B		100(46)
2	**42a**	MeOH	20/18	B	**43a**	100(96)
3		H_2_O	20/22	B		32(5)
4		MeCN	20/2	A		100(89)
5	**42b**	MeOH	20/3	A	**43b**	100(71)
6		H_2_O	20/3	A		88(68)
7		MeCN		B		0
8	**42c**	MeOH		B	**43c**	60(38)
9		H_2_O		B		93(17)

^a^ Ratio of **42** / I_2_ / **1** : A = 1 / 0.5 / 0.6; B = 1 / 1 / 1; ^b^ The first value is the conversion of starting material, the values in parentheses are the yield of isolated **43**.

### 2.3. Electrophilic Functionalization of Arenes Using Anionic Precursors other than Halogens

It was demonstrated that various anionic precursors could be oxidized by **1** to active electrophilic species which efficiently functionalized the benzene ring. As already mentioned, bromide and chloride anions are readily oxidized to their electrophile equivalents and the same was established for thiocyanate (CNS^−^) and nitrite (NO_2_^−^) anions, which were transformed by **1** into CNS^+^ and NO_2_^+^ species, respectively, and efficiently functionalized electron-rich benzene derivatives [42]. Anions such as ACO^−^ or TfO^−^ were found to be relatively resistant towards oxidation with **1**, while cyanide, cyanate, methoxide or thiomethoxide anions could not be oxidized with **1** at all.

### 2.4. Functionalisation at a Benzylic Carbon Atom

In the transformations described in sections **2.2** and **2.3** F-TEDA-BF_4_ acts as an oxidant forming electrophilic species from various unreactive sources which afterwards collapse with the electron-rich part of the organic substrates. In this section the opposite situation is described and a variety of examples reviewed where **1** acted as oxidant for the chosen substrates, thus forming an electron deficient reactive intermediate which reacted with an external nucleophile. 

An example of this kind is the versatile derivatisation of a benzylic carbon atom in hexamethylbenzene (HMB, **44**). Table 3 summarizes reactions of HMB with F-TEDA-BF_4_ in the presence of alcohols or potassium salts of perfluoroalkanoic acids in MeCN media. Pentamethyl-benzylalkyl ethers (entries 1-9) or esters (entries 10-15) were readily obtained in high to excellent yields. When this reaction was performed in TFA in the presence of various nitriles, Ritter-type benzylic amidation took place and the corresponding pentamethylbenzyl amides (**46**, Table 4) were formed in high yield [44]. 

**Scheme 6:**
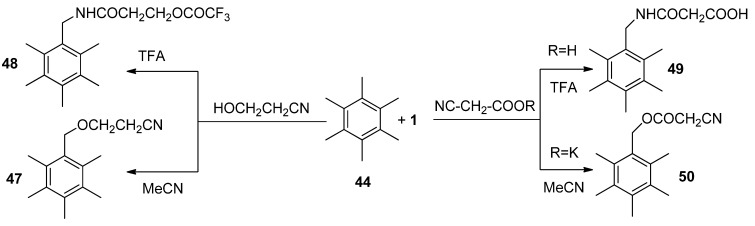
F-TEDA-BF_4_ mediated benzylic functionalisation of hexamethyl benzene in the presence of compounds bearing two different nucleophilic centres.

Using appropriate reaction conditions, selective functionalisation of HMB can be obtained in the presence of compounds bearing two different nucleophilic active sites. Reaction in MeCN in the presence of 2-cyanoethanol gave the benzylic ether derivative (**47**, Scheme 6), while in TFA Ritter transformation took place and benzyl amide derivative **48** was formed.

**Table 3:** Reactions of hexamethyl benzene **44** with F-TEDA-BF_4_**1** in the presence of alcohols or potassium salts of carboxylic acids.^a^ 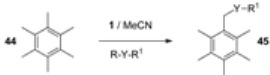

Entry	R	Y	R ^1^	Yield (%)	Reference
1	H	O	*i-*Pr	88	[44]
2	H	O	*n*-hexyl	90	[44]
3	H	O	*c*-pentyl	98	[44]
4	H	O	Bn	75	[44]
5	H	O	MeOCH_2_CH_2_	93	[44]
6	H	O	CF_3_CH_2_	75	[44]
7	H	O	CF_3_CF_2_CH_2_	70	[45]
8	H	O	CF_3_(CF_2_)_2_CH_2_	70	[45]
9	H	O	(CF_3_)_2_CH	71	[45]
10	H	OCO	Me ^b^	97	[44]
11	H	OCO	CF_3_^b^	97	[45]
12	K	OCO	CF_3_CF_2_	97	[45]
13	K	OCO	CF_3_CF_2_CF_2_	72	[45]
14	K	OCO	CF_2_(CF_2_)_3_CF_2_	96	[45]
15	K	OCO	CF_3_(CF_2_)_5_CF_2_	90	[45]

^a^ Reaction conditions: HMB (2 mmol), F-TEDA-BF_4_ (2.2 mmols), 25 mmol of R^1^OH or 2.4 mmol of KOCOR^1^, MeCN (20 mL), 55 °C, 1–2 hours. ^b^ Reactions were performed in AcOH or TFA, respectively. as solvent.

**Table 4:** Ritter-type functionalization of the benzylic position in hexamethylbenzene mediated by F-TEDA-BF_4_ [44].^a^ 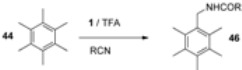

Entry	R	Time (h)	Yield
1	Et	2	82
2	*n*-pentyl	3	65
3	*i*-Pr	2	75
4	*c*-Pr	1	86
5	MeOCH_2_	1	95
6	MeOCOCH_2_	1	98
7	EtOCOCH_2_	1	84
8	Ph	1	75
9	*p*-COOMe-Ph	1	71
10	Bn	1	90
11	C_6_F_5_	1	81

^a^ Reaction conditions: HMB (5 mmol), RCN (15 mmol), F-TEDA-BF_4_ (5 mmol) TFA (50 mL), 55 °C.

Similarly, cyanoacetic acid as a source of an external nucleophile was activated at its cyanide moiety if TFA was used as solvent and the corresponding benzyl amide **49** was formed, while in MeCN, potassium cyanoacetate acted as a carboxy nucleophile and pentamethylbenzyl cyanoacetate **50** was formed [44].

**Scheme 7:**
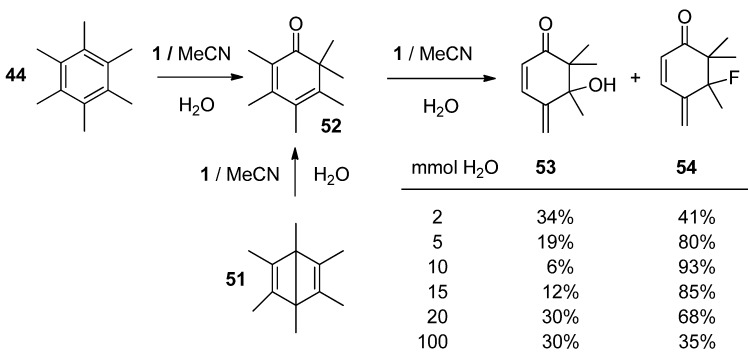
Transformation of hexamethyl benzene with F-TEDA-BF_4_ in the presence of water.

A quite different course of reaction of HMB with **1** was established in the case when water was used as the external nucleophile. In aqueous MeCN phenyl ring transformation took place, starting with *ipso* attack of water and further rearrangement of the methyl group as the main process. Primarily formed rearranged 2,3,4,5,6,6-hexamethylcyclohexa-2,4-dienone (**52**, Scheme 7) was further transformed to 5-hydroxy-2,3,5,6,6-pentamethyl-4-methylenecyclohex-2-en-1-one **53** or 5-fluoro-2,3,5,6,6-pentamethyl-4-methylenecyclohex-2-en-1-one **54**; the relative yield of these final products was found to be dependent on the concentration of water in the reaction mixture (Scheme 7). Product **52** was independently obtained in excellent yield by treating hexamethyl Dewar benzene **51** with an aqueous MeCN solution of **1**. In the presence of water and alcohol as the second external nucleophile, competition between ring and benzylic functionalisation was observed. In the case of MeOH or EtOH up to 40% of benzylic functionalisation took place thus forming benzyl alkyl ethers, while in the presence of trifluoroethanole or hexafluoro i-propanole product **54** was selectively formed in excellent yield [46]. 

The reaction of 1,2,4,5-tetramethyl benzene (**55**, Table 5) with **1** was also studied and the role of solvent and external nucleophile on the course of the transformation established. In MeOH benzylic functionalisation forming benzyl methyl ether derivative **57a** (entry 1, Table 5) was the exclusive process, in acetic acid ring attack of the nucleophile forming 2,3,5,6-tetramethylphenyl acetate (**58a**, entry 2) was found to be predominant process, while in TFA exclusive ring esterification thus forming 2,3,5,6-tetramethylphenyl trifluoroacetate **58b** (entry 3) was observed. In reactions performed in MeCN, the nature of the external nucleophile regulated the course of reaction. In the presence of TFA (entry 4) Ritter-type benzylic functionalization to *N*-(2,4,5-trimethylbenzyl)acetamide **56** took place exclusively, in the presence of acetic acid benzylic amidation, benzylic and ring acetoxylation competed, while in the presence of water (entry 6) *ipso* attack of water followed by methyl group rearrangement and further fluorination or fluoro amidation forming equal amounts of products **59** and **60** was observed [46]. Other isomeric tetra- and trimethyl benzene derivatives were also tested in the presence of **1** and an external nucleophile; the kinetics of the reactions of polymethyl-substituted benzene derivatives with **1** studied and the results obtained supported the assumption that single electron transfer (SET) is the dominant process in these transformations [46].

**Table 5:** Effect of solvent and external nucleophile on the transformation of 1,2,4,5-tetramethyl benzene with F-TEDA-BF_4_. ^a^ 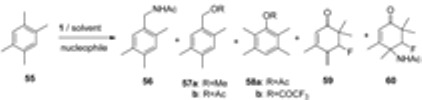

		Relative ratio of products (%)					
Entry	Solvent/nucleophile	56	57	58	59	60	Yield (%) ^b^
1	MeOH / -	-	100	-	-	-	93
2	AcOH / -	-	29	71	-	-	85
3	TFA / -	-	-	100	-	-	95
4	MeCN / TFA ^c^	100	-	-	-	-	82
5	MeCN / AcOH	27	21	52	-	-	80
6	MeCN / H_2_O ^d^	-	-	-	50	50	95

^a^ Reaction conditions: 1,2,4,5-tetramethyl benzene (1 mmol), F-TEDA-BF_4_ (1 mmol), 10 mL of solvent and 10 mmol of nucleophile, 60–120 °C, 1.5–18 hours; ^b^ Total yield of products calculated on starting material; 10 mL of MeCN/TFA = 9/1; ^c^ 2 mmols of **1** was necessary for total conversion of **55**.

### 2.5. Lewis Acid-Type Mediation of Condensation Reactions and Ring Opening of Epoxides

**Scheme 8:**
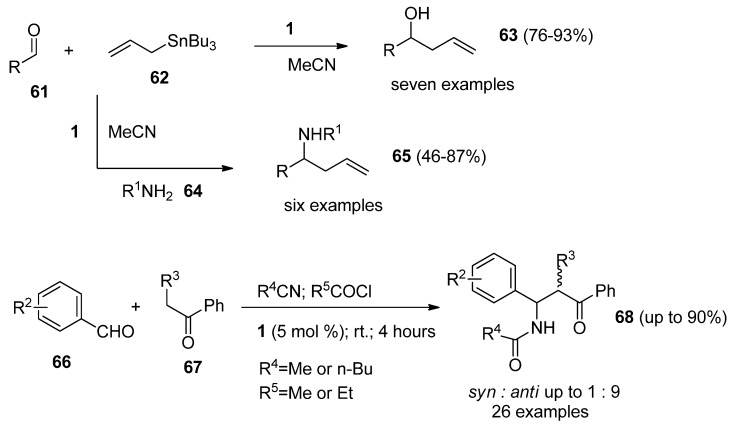
Synthesis of homoallylic alcohols or amines and *β-*acetamido ketones mediated by F-TEDA-BF_4_.

*Selectfluor F-TEDA-BF_4_* can act as a Lewis acid and this fact was used to advantage in a variety of condensation reactions. Reactions of aryl or alkyl adehydes (**61**, Scheme 8) with allylbutyltin mediated **62** by **1** in MeCN resulted in the formation of homoallylic alcohols **63**, and the analogous reactions in the presence of amines **64** lead to homoallylic amines **65** in good yields with excellent moisture and air tolerance [47]. 

An efficient, room temperature process for the stereoselective synthesis of β-amido ketones (**68**, Scheme 8) employing a one-pot multi-component reaction of benzaldehyde derivatives **66**, alkyl phenyl ketone **67**, an acid chloride, and a nitrile in the presence of catalytic amounts of F-TEDA-BF_4_ was reported [48]. The method offers advantages such as high yield, short reaction time and energy efficiency, high *anti*-stereoselectivity and a simple work-up protocol.

**Scheme 9:**
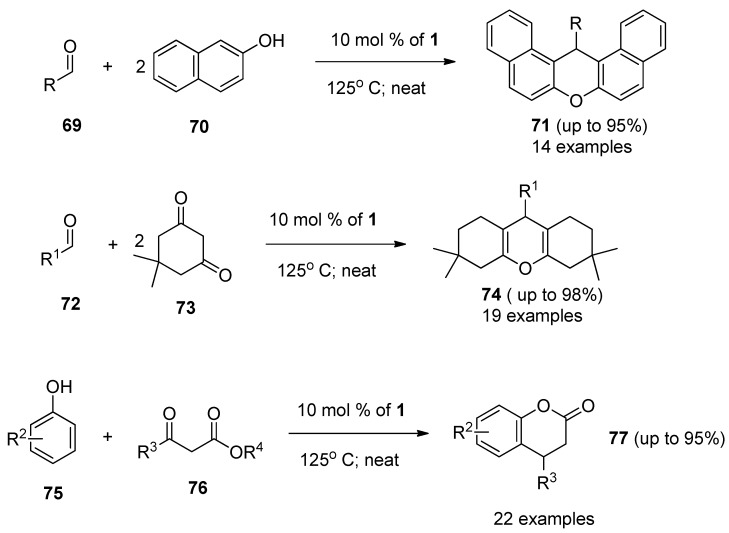
F-TEDA-BF_4_ catalyzed condensation reactions forming oxygen heterocycles.

A synthetic protocol for the preparation of aryl-*14H*-dibenzo[*a,j*]xanthene derivatives (**71**, Scheme 9) through the F-TEDA-BF_4_ catalyzed one-pot condensation of substituted benzaldehydes **69** with 2-naphthole **70** under solvent-free conditions was devised and methodology efficiently demonstrated by 14 examples [49]. An efficient procedure for the synthesis of 1,8-dioxo-octahydro-xanthenes **74** through one-pot condensation of 5,5-dimethyl-1,3-cyclohexadione **73** with aryl aldehyde derivatives **72** in the presence of catalytic amounts of **1** was developed and efficiently demonstrated with 19 examples [50]. One-pot condensation of β-ketoesters **76** and substituted phenols **75** catalyzed by **1** resulted in the efficient formation of 2H-chromen-2-one derivatives 77 [51]. Reactions were performed under solvent-free conditions and application of ultrasonic irradiation improved the yields and reduced the reaction times [52]. 

**Scheme 10:**
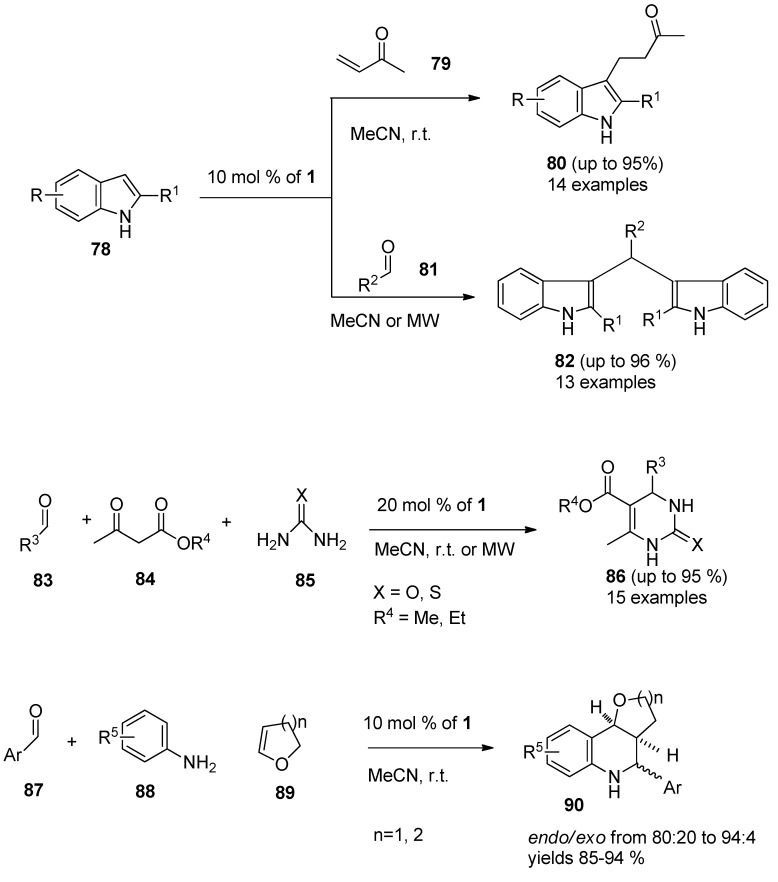
F-TEDA-BF_4_ catalyzed condensation reactions forming nitrogen heterocycles.

It was also found that F-TEDA-BF_4_ efficiently catalyzed the conjugate addition of indoles (**78**, Scheme 10) with α,β-unsaturated ketones **79** thus forming Michael adducts **80** under extremely mild reaction conditions and the methodology was confirmed with 14 examples [53]. The same approach was used in the case of reactions of indoles 78 with different aldehydes **81**, resulting in the formation of bis(indolyl)methane derivatives **82** and the efficiency of the reaction was improved by MW irradiation under solvent-free conditions [54]. The Biginelli reaction, *i.e.*, one-pot multi-component condensation of aldehyde **83**, β-ketoester **84** and urea or thiourea **85** forming dihydropyrimidinones **86**, was considerably improved when 1 was used as the catalyst [55]. Aryl imines formed *in situ* from aryl aldehydes **87** and aromatic amines **88** underwent smooth [4+2] cycloaddition reactions with cyclic enol ethers **89** such as 3,4-dihydro-2H-pyran or 2,3-dihydrofuran in the presence of 10 mol % **1** in MeCN at room temperature to afford pyrano- and furanotetrahydroquinoline derivatives **90** with high *endo*-stereoselectivity and high yield [56].

**Scheme 11:**
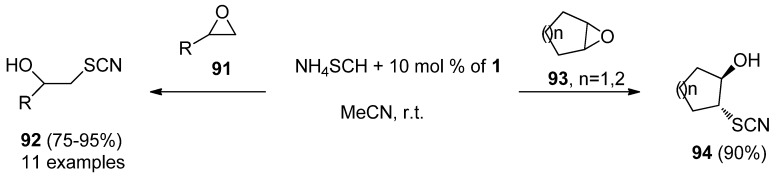
Regio and stereoselective ring opening of epoxides catalysed by F-TEDA-BF_4_.

A variety of epoxides (**91**, **93**, Scheme 11) could be efficiently opened regio and stereoselectively with ammonium thiocyanate in the presence of 10 mol% of F-TEDA-BF_4_ in MeCN at room temperature, affording the corresponding β-hydroxy thiocyanates **92**, in the case of cyclic epoxides with *trans* stereochemistry **94** [57]. 

### 2.6. Deprotection of Functional Groups

**Scheme 12:**
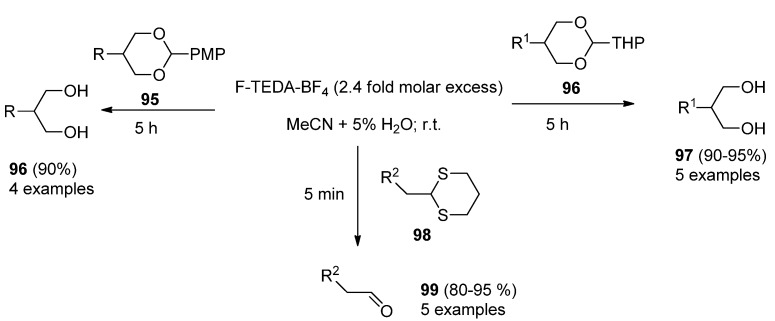
Cleavage of PMP, THP, and 1,3-dithiane protecting groups by F-TEDA-BF_4_.

An efficient method for cleavage of *p*-methoxybenzylidene (PMP), tetrahydropyranyl (THP) and 1,3 dithiane protecting groups with F-TEDA-BF_4_ was reported. PMP and THP are very useful protecting groups for diols, but their deprotection usually demands strong acidic or oxidative conditions, and 1,3-dithiane deprotection usually requires harsh conditions, too, which is inconvenient in the case of multifunctionally derivatized target molecules. It has been shown that **1** can smoothly and efficiently cleave PMP (**95**, Scheme 12), THP **97** or 1,3-dithiane protected compounds under mild reaction conditions [58]. 

**Scheme 13:**
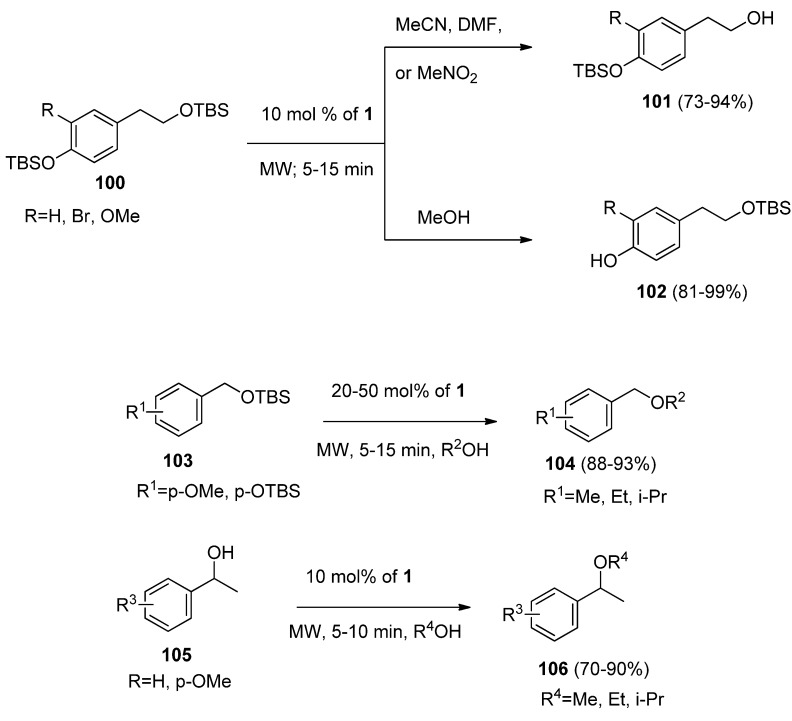
Chemoselective microwave-assisted deprotection of alkyl and aryl silyl ethers, transetherification and etherification of benzylic hydroxyl groups catalyzed by F-TEDA-BF_4_.

A novel microwave-assisted, chemoselective and efficient method for the cleavage of aliphatic and aromatic silyl ethers catalyzed by F-TEDA-BF_4_ was reported. A wide range of aliphatic and aromatic *tert-*butyldimethyl (TBS) protected silyl ethers (**100**, Scheme 13) were chemoselectively cleaved. In MeCN, MeNO_2_ or DMF alkyl silyl ether was deprotected (**101**), while in MeOH phenolic silyl ether was cleaved (**102**). In addition, the transetherification of benzylic TBS-protected ethers **103** and etherification of benzyl alcohols **105** in alcoholic solvents resulting in the formation of **104** or **106** was observed [59].

### 2.7. Transformations of Halogen-Substituted Azabicyclic Compounds

Stereoselective synthesis of 5,6-difunctionalized-2-azabicyclo[2.1.1]hexanes containing 5*-anti*-fluoro or hydroxyl in one methano bridge have been prepared by the F-TEDA-BF_4_ mediated rearrangement of derivatives of N-alkoxycarbonyl-6-*exo*-iodo-2-azabicyclo[2.2.0]hexanes [60]. It was also found that **1** has the ability to act as a nucleofuge for hydrolysis of β-*anti*-halides in N-alkoxycarbonyl derivatives of 6*-anti*-Y-7-*anti*-X-2-azabicyclo[2.2.1]heptanes (**107**, Table 6) and 4-*anti*-Y-8-*anti*-X-6-azabicyclo[3.2.1]octanes (**109**, Table 7), thus forming hydroxyl substituted derivatives **108** or hydroxyl or oxo-substituted products **110**, respectively [61].

**Table 6:** Hydrolysis of β-halo-N-aloxycarbonyl-2-azabicyclo[2.2.1]heptanes with F-TEDA-BF_4_. 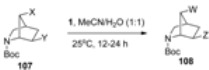

Entry	X	Y	W	Z	Yield (%)
1	Br	Br	Br	OH	60
2	I	Cl	I	OH	35
3	I	OH	OH	OH	80
4	I	F	OH	F	86

**Table 7:** F-TEDA-BF_4_ as a nucleofuge and oxidant of β-halo-N-Aloxycarbonyl-2-azabicyclo[3.2.1]octanes. 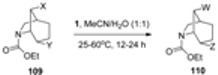

Entry	X	Y	W	Z	Yield (%)
1	Br	Br	Br	=O	91
2	Br	OH	Br	=O	99
3	I	Cl	I	OH	77
4	I	OH	I	=O	20

### 2.8. Functionalization of N-Heterocycles

The direct thiolation of indoles (**111**, Table 8) with a variety of thiols **112** has been achieved in the presence of F-TEDA-BF_4_. This versatile and efficient method works for thiolation of 5- or 7-substituted indoles, as well as for 1-substituted (entries 6 and 9) and 2-substituted (entries 5, 12, and 13) indole derivatives with aromatic thiols (entries 1–17), alkyl thiols (entries 18 and 19) and benzyl thiol (entry 20). The reaction protocol is simple; the transformation goes to completion at room temperature within 20–30 minutes, efficiently and selectively forming 3-sulfenylindoles **113** [62]. 

**Table 8:** F-TEDA-BF_4_ mediated synthesis of 3-sulfenylindoles. 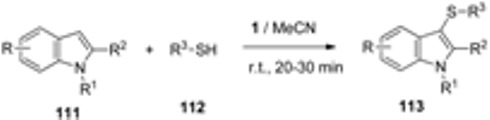

Entry	R	R ^1^	R ^2^	R ^3^	Yield [%]
1	H	H	H	Ph	96
2	5-Br	H	H	Ph	85
3	5-OMe	H	H	Ph	96
4	7-Et	H	H	Ph	89
5	H	H	Me	Ph	89
6	H	Bn	H	Ph	87
7	H	H	H	4-Cl-Ph	92
8	5-OMe	H	H	4-Cl-Ph	97
9	H	Bn	H	4-Cl-Ph	93
10	7-Et	H	H	4-Cl-Ph	90
11	7-Et	H	H	4-Me-Ph	89
12	H	H	Me	4-Cl-Ph	94
13	H	H	H	4-Cl-Ph	94
14	5-Br	H	H	4-Me-Ph	90
15	H	H	H	4-NO_2_-Ph	78
16	H	H	H	4-Br-Ph	87
17	H	H	H	2-naphthyl	85
18	H	H	H	Et	87
19	5-Br	H	H	*n*-Bu	78
20	H	H	H	Bn	82

Various substituted indoles **111** have been efficiently thiocyanated under mild and neutral conditions to selectively produce 3-indoylthiocyanates **114** (Table 9) in excellent yield following the reaction of indole derivatives with ammonium thiocyanate in the presence of F-TEDA-BF_4_. Mechanistically, the reaction was declared to be the electrophilic substitution of indole derivatives by in situ generated thiocyanogen electrophilic species from **1** and ammonium thiocyanate. Following the same protocol was also successful for thiocyanation of azaindole, carbazole and pyrrole [63]. 

**Scheme 14:**
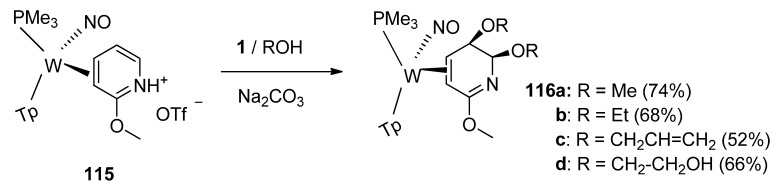
Dimethoxylation of η^2^-pyridinium complex mediated by F-TEDA-BF_4_.

The tungsten η^2^-coordinated pyridinium complex **115** (Scheme 14) undergoes a stereoselective dialkoxylation when treated with F-TEDA-BF_4_ in alcohol. The alkoxy groups add to the 5-and 6-positions of TpW(NO)(PMe_3_)(3,4-η^2^-methoxypyridine **115** in a *syn* fashion. The reaction pathway has been not completely investigated but apparent stabilization by tungsten of the allyl cation intermediate resulting from the electrophilic attack of **1** to the 5,6-double bond on **115**, captured by alkoxy anion and further fluorine atom replacement by the alkoxide in a subsequent substitution reaction resulting in the final formation of **116** seems to be a reasonable explanation of the reaction route [64]. 

**Table 9:** Thiocyanation of indole derivatives with ammonium thiocyanate using F-TEDA-BF_4_. 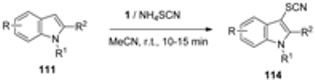

Entry	R	R ^1^	R ^2^	Yield [%]
1	H	H	H	95
2	H	H	Me	92
3	7-Et	H	H	94
4	5-NO_2_	H	H	93
5	5-CN	H	H	92
6	5-Br	H	H	93
7	5-OMe	H	H	96
8	H	H	Ph	89
9	H	Bn	H	94
10	H	Bn	Ph	86

### 2.9. Gold-Catalyzed and Palladium-Catalyzed Oxidative C-C or C-Heteroatom Bond Formation

Cross-coupling reactions are powerful tools for the rapid construction of organic molecules and one of the most important and valuable approaches in organic synthesis. Various transition metals catalyze these valuable transformations and gold was introduced for this purpose recently [65]. The gold/Selectfluor F-TEDA-BF_4_ tandem was recognized as a valuable combination in numerous cross-coupling C-C or C-heteroatom bond formations. 

The pioneer work on this area has been done by Zhang and co-workers with the discovery that under oxidative conditions gold catalyzes the coupling of propargyl acetates (**117**, Table 10) with boronic acids **118** resulting in the formation of α-aryl α,β-enones **119** in moderate to good yields and total *E*-stereoselectivity [66]. Following the proposed mechanism, reactions start by gold mediated 3,3-rearangement of propargyl acetates to allenyl acetates and their hydrolysation into the vinyl-Au(I) species which is subsequently oxidized by F-TEDA-BF_4_ to furnish Au(III) intermediates; later these undergo transmetallation with boronic acids to give diorganogold derivatives, which after reductive elimination, regenerate the active Au(I) species and deliver the final cross-coupled products **119**. Without the presence of boronic acid derivatives, oxidative dimerization of propargylic acetates was observed [67].

**Table 10:** Gold-catalyzed oxidative cross-coupling of propargyl acetates with boronic acids. 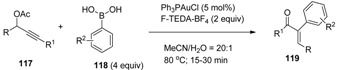

Entry	R	R ^1^	R ^2^	Yield (%)
1	Ph	*n*-butyl	H	62
2	*i*Pr	*n*-butyl	H	65
3	Me	Ph	H	59
4	Me	MeOCH_2_CH_2_	H	60
5	Me	cyclohexyl	H	68
6	cyclohexyl	cyclohexyl	H	70
7	PhCH_2_CH_2_	*n*-butyl	H	70
8	4-Br-Ph	*n*-butyl	H	59
9	AcOCH_2_CH_2_	*n*-butyl	H	61
10	H	cyclohexyl	H	61
11	cyclohexyl	*n*-butyl	4-Me-Ph	72
12	cyclohexyl	*n*-butyl	4-CO_2_Me-Ph	57
13	cyclohexyl	*n*-butyl	4-Cl-Ph	58
14	cyclohexyl	*n*-butyl	3-CO_2_Me-Ph	45

**Scheme 15:**
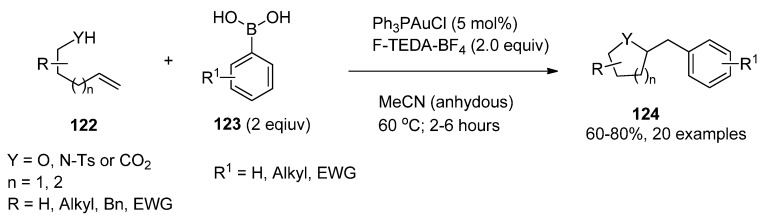
Gold-catalyzed oxidative carboheterofunctionalization of alkenes.

Analogous reactions were observed when propargyl benzoates (**120**, Table 11) were treated under similar reaction conditions and 1-benzoylvinyl ketones **121** were isolated [68]. Intramolecular cross-coupling resulting in carboamination, carboalkoxylation or carbolactonization processes and formation of N- or O-heterocycles (**123**, Scheme 15) were reported when alkenes bearing a terminal hydroxyl, tosylamido or carboxy group (**122**) were treated with the gold_cat_ /F-TEDA-BF_4_ tandem in the presence of boronic acid [69]. The scope of this reaction was considerably extended using bimetallic gold complexes as catalysts. The best results were obtained in the case of [dppm(AuBr)_2_] catalyst where bis(diphenylphosphine)methane (dppm) was the ligand part of the bimetallic Au catalyst and a variety of alkenes and boronic acid reactants cross-coupled forming N-heterocycle derivatives [70].

**Scheme 16:**
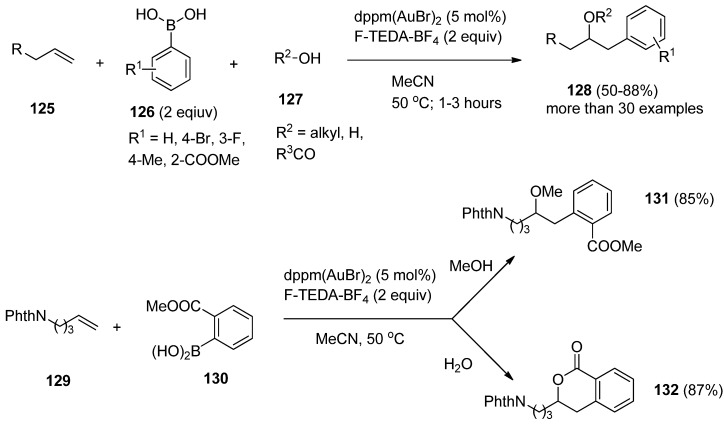
Gold-catalyzed F-TEDA-BF_4_ mediated oxyarylation of alkenes.

The same group of authors further reported three-component coupling reactions using this valuable methodology. Various combinations of alkenes (**125**, Scheme 16), boronic acid derivatives **126**, and alcohols, carbocyclic acids or even water (**127**) were treated with catalytic amounts of dppm(AuBr)_2_ bimetallic complex in the presence of F-TEDA-BF_4_ and oxyarylation of the double bond took place resulting in compounds **128**. The ability to use either alcohols or water as nucleophiles in this gold-catalyzed three-component coupling provided access to a greater diversity of products. In the case of alkene **129** and 2-carboxymethyl boronic acid **130**, methoxyarylation producing **131** took place when methanol was used as nucleophile, while in the presence of water, hydroxyarylation, followed by *in situ* lactone formation **131** was the result of the reaction [71].

**Table 11:** Gold-catalyzed synthesis of 1-benzoylvinyl ketones from propargylbenzoates. 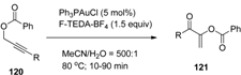

Entry	R	Yield (%)
1	cyclohexyl	76
2	Ph	66
3	cyclopropyl	56
4	BnOCH_2_CH_2_	71
5	BzOCH_2_CH_2_	78
6	BzCH_2_CH_2_CH_2_	70

The versatility of this methodology was expanded and arylsilicon compounds were taken as transmetallation components. The best results were obtained with phenyltrimethylsilane (**133**, Table 12) and efficient three-component coupling was accomplished when alkene **129**, various alcohols and **133** were treated with the dppm(AuBr)_2_ / F-TEDA-BF_4_ tandem, resulting in oxyarylated products **134**. As in the case of boronic acid in the presence of methanol, 2-carboxymethyl-trimethylphenylsilane was methoxyarylated to product **131**, while the water mediated reaction yielded lactone product **132**. In the case when a side chain bearing terminal alkene functionality is bonded at the *ortho* position of phenyltrimethylsilane reagent (**135**, Table 13), intramolecular coupling reaction took place resulting in products **136** [72].

**Table 12:** Gold-catalyzed and F-TEDA-BF_4_ mediated three-component oxyarylation of C-C double bond. 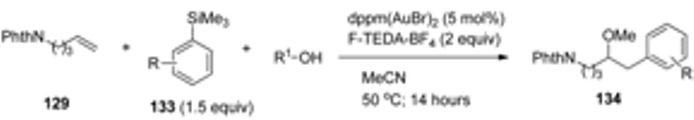

Entry	R	R ^1^	Yield [%]
1	4-OAc	Me	83
2	4-OTf	Me	53
3	4-N(Me)Ts	Me	66
4	4-Me	Me	73
5	4-Br	Me	82
6	4-CHO	Me	77
7	4-CO_2_Me	Me	68
8	3-CO_2_Me	Me	83
9	2-CH_2_CH_2_OH	Me	69
10	H	Me	87
11	H	Et	83
12	H	*i*-Pr	81
13	H	*t*-Bu	37
14	H	neopentyl	64
15	H	cyclopentyl	68
16	H	2-methoxyethyl	86
17	H	H	77
18	2-CH_2_CH_2_OH	H	55

A comparison of gold-catalyzed oxyarylation of terminal alkenes (**137**, Table 14) using arylsilanes **138a** or arylboronic acids **138b** as transmetallating reactants was reported. The results collected in Table 14 demonstrate some advantages of the application of arylboronic acids in these reactions but the differences are not so remarkable. The commercially available gold catalyst Ph_3_PAuCl was used, making this valuable and versatile transformation even more attractive [73].

**Table 13:** Gold-catalyzed and F-TEDA-BF_4_ mediated intramolecular coupling reactions. 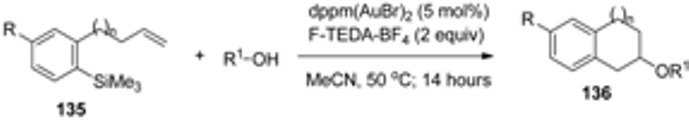

Entry	R	R ^1^	n	Yield (%)
1	H	H	1	66
2	H	Me	1	73
3	H	Et	1	70
4	H	H	0	15
5	F	H	1	47
6	F	Et	1	68
7	Cl	H	1	62
8	Cl	Me	1	65
9	CF_3_	H	1	51
10	CF_3_	Me	1	59
11	Ph	Me	1	74

**Scheme 17:**
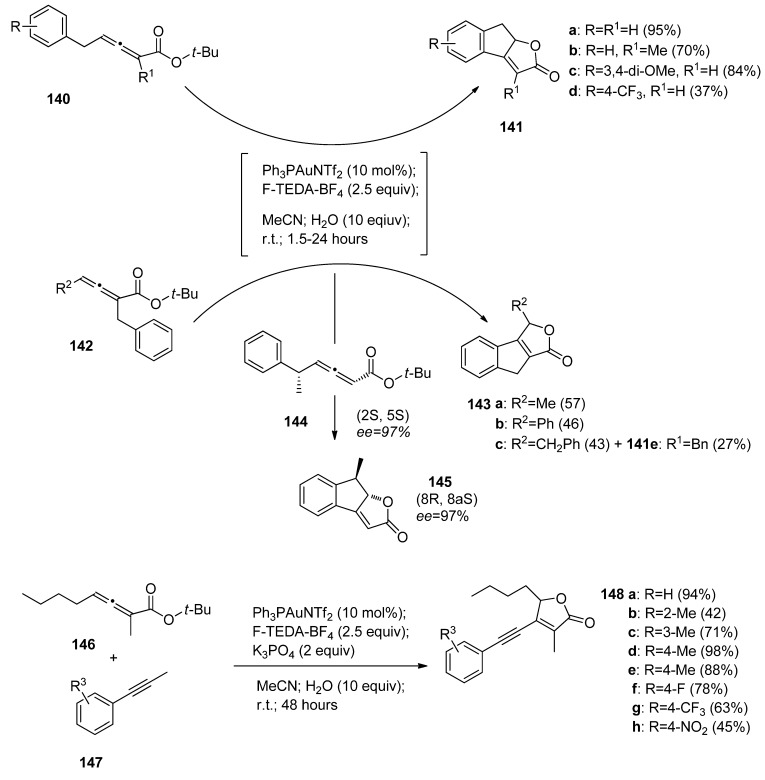
Gold-catalyzed F-TEDA-BF_4_ mediated oxidative transformations of allenoates.

Another valuable application of the Au(catalyst)/F-TEDA-BF_4_(oxidant) tandem was reported by Gouverneur and co-authors. They developed a novel cascade cyclization cross-coupling process leading to tricyclic dihydroindenofurane-type compounds (**141a-e**, **143a-c**, and **145**, Scheme 17) following the Ph_3_PAuNTf_2_ catalyzed and F-TEDA-BF_4_ mediated transformations of t-butyl ester substituted allenoates bearing a benzyl functional group on the opposite side of an allenoate moiety (**140**), or vicinal to a *tert*-butyl ester group (**142**). The substrates **140** readily gave products **141a-e**, while starting materials **142** gave products **143 a–b**. In the case when both relevant allenoate carbon atoms were substituted by a benzyl group, the formation of product **143c** was found to be preferential. It has also been established that the transformation is stereospecific, since pure enantiomer **144** gave only enantiomer **145** [74]. 

The same group of authors developed efficient cascade cyclization-oxidative alkynyliation of allenoates (**146**, Scheme 17) with phenyl acetylenes **147**, resulting in the formation of 5-butynyl-3-methyl-4-(phenylenthynyl)furan-2(5*H*)-one derivatives **148**. The selectivity as well as the efficiency of the transformation decreased if other than a n-butyl group was bonded to alleonate **146**, or an alkyl group bonded to the alkynyl substrate **147** [75].

Various arylgold(I) and alkynylgold(I) triphenylphosphane complexes (**149**, Table 15) were subjected to electrophilic halogenations reagents. Iodo, bromo and chloro reagents gave halogenated products, while reactions with F-TEDA-BF_4_ followed exclusively the homocoupling process and corresponding dimeric products **150** were isolated in high yield [76].

**Scheme 18:**
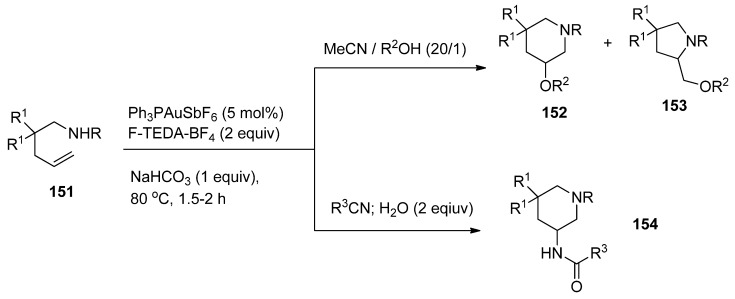
Gold-catalyzed and F-TEDA-BF_4_ assisted aminooxygenation or aminoamidation of unactivated alkenes.

Aminooxygenation of unactivated alkenes (**151**, Scheme 18) were achieved by gold catalysis assisted by F-TEDA-BF_4_ as an oxidant. In the case when the solvent was 20/1 mixture of MeCN and water (R^2^ = H), methanol (R^2^ = Me), or ethanol (R^2^ = Et) mixtures of piperidine **152** and pyrrolidine derivatives **153** were formed. The formation of piperidine derivatives prevailed. On the other hand, by reducing the amount of water in the reaction mixture to only 2 equivalents and using nitriles as the reaction media, the aminoamidation process took place and 3-amido substituted piperidine derivatives **154** were selectively formed [77].

**Table 14:** Gold-catalyzed and F-TEDA-BF_4_ mediated oxyarylation of terminal alkenes using arylsilanes [73] or arylboronic acids [71]. 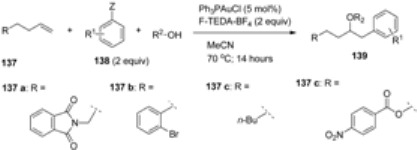

				Yield [%]	
Entry	Alkene	R ^1^	R ^2^	Z = SiMe_3_	Z = B(OH)_2_
1	**137 a**	H	Me	71	79
2	**137 a**	H	Et	69	85
3	**137 a**	H	*i*-Pr	70	90
4	**137 a**	H	*t-*Bu	-	33
5	**137 a**	H	neopentyl	80	91
6	**137 a**	H	*c*-pentyl	57	85
7	**137 a**	H	Ac	79	62
8	**137 a**	4-Me	Me	55	88
9	**137 a**	2-Me	Me	20	-
10	**137 a**	4-Br	Me	80	90
11	**137 a**	3-F	Me	63	79
12	**137 a**	4-CO_2_Me	Me	80	83
13	**137 b**	4-Br	*c*-pentyl	51	69
14	**137 b**	4-Br	Ac	51	51
15	**137 b**	H	H	76	76
16	**137 c**	4-Br	*c*-pentyl	38	76
17	**137 c**	4-Br	neopentyl	85	73
18	**137 c**	H	H	78	73
19	**137 d**	H	H	75	67

Recently the Zhang group reported the first oxidative cross-coupling reaction between an aryl C-H bond and an alkyl gold compound generated *in situ*, combining Au(I)/Au(III) catalysis with C-H functionalization. They have chosen *N,N*-diallyl-*N´*-phenylurea derivatives (**155d-k**, Table 16) as a substrates, (4-CF_3_-C_6_H_4_)_3_P-Au-NTf_2_ as the catalyst, and F-TEDA-BF_4_ as the oxidant and following an initial aminoauration and subsequent intramolecular [3+2] annulation process isolated tricyclic indoline derivatives **156** in high yield. The efficiency of the reaction was significantly improved by the addition of 30 eqiuvalents of water in TFH as the optimal reaction media and the transformation was successful in the case when the additional allyl group in **155** was replaced by benzyl (entry 1), alkyl (entry 2) or phenyl group (entry 3). On the basis of performed deuterium labeling and kinetic isotope effect studies along with the isolation of alkyl gold intermediates the reaction mechanism anticipating an electrophilic aromatic substitution for the C-H functionalization and a subsequent inner-sphere concerted reductive elimination for the C_sp2_-C_sp3_ bond formation were strongly supported [78].

**Table 15:** Homocoupling reactions of organogold(I) triphenylphosphane compounds induced by F-TEDA-BF_4_. 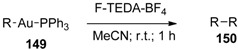

Entry	R	Yield (%)
1	Ph	90
2	3-nitrophenyl	91
3	3-methoxyphenyl	85
4	4-methoxyphenyl	94
5	2-formylfuran-5-yl	82
6	3-formylfuran-5-yl	81
7	phenyletynyl	94
8	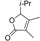	71

**Table 16:** Gold-catalyzed and F-TEDA-BF_4_ mediated C-C coupling through C-H functionalization. 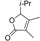

Entry	**155**	R	R ^1^	Yield [%] of 156
1	**a**	H	Bn	75
2	**b**	H	*n*-hexyl	69
3	**c**	H	Ph	70
4	**d**	4-Me	allyl	72
5	**e**	2-Me	allyl	43
6	**f**	3-Me	allyl	79
7	**g**	4-F	allyl	70
8	**h**	4-OTs	allyl	67
9	**i**	4-CF_3_	allyl	64
10	**j**	4-COOEt	allyl	84
11	**k**	4-Ac	allyl	75

**Scheme 19:**
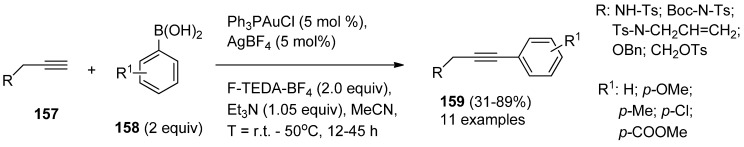
Gold-catalyzed F-TEDA-BF_4_ mediated Sonogashira-type cross-coupling reactions of terminal alkynes with arylboronic acids.

In the same laboratory a straightforward, efficient, and reliable catalyst system for the Sonogashira cross-coupling reaction of terminal alkyne derivatives (**157**, Scheme 19) with arylboronic acids **158** was developed very recently. The catalyst consisting Ph_3_PAuCl and AgBF_4_ gave the best results in the presence of F-TEDA-BF_4_ as the oxidant and Et_3_N as the base and the scope of the method was illustrated by eleven examples of cross-coupling yielding aryl functionalized alkyne derivatives **159** [79].

**Scheme 20:**
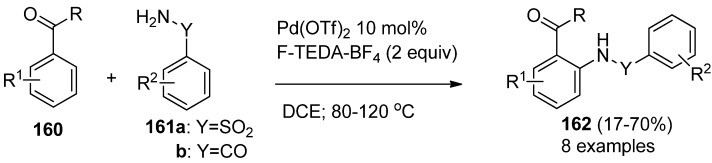
Palladium-catalyzed F-TEDA-BF_4_ mediated *ortho* amidation of aromatic ketones.

Palladium-catalyzed directed *ortho* amidation of aromatic ketones (**160**, Scheme 20) with both sulfoanamides **161a** and amides **161b** has been accomplished using different oxidants, including N-F compounds. The efficiency of the formation of the corresponding sulfonamides **162a** or amides **162b** was moderate to good when F-TEDA-BF_4_ mediated the reactions. It has been proposed and supported by X-ray crystallography that the formation of cyclopalladation complexes of aryl ketones and amides are the key intermediates for this valuable transformation. The palladium(II) complex is oxidized to the Pd(IV) moiety, which following reductive elimination, ends in the final ortho amido derivatized product [79]. 

## 3. Conclusions and Perspectives

*Selectfluor^TM^ F-TEDA-BF_4_* is one of the most popular electrophilic fluorination reagents. Besides this, its major role in organic synthesis, it also acts as a reagent or catalyst of many functionalizations of organic compounds other than fluorinations, where its characteristics as an oxidant or a Lewis acid regulate the versatile utility. As a transformer of oxidizable functional groups F-TEDA-BF_4_ could be very efficient but from the green chemical point of view its perspectives, except for specific cases, are limited, as well as in the field of oxidative halogenations, where a variety of greener protocols using environmentally more acceptable oxidants, such as H_2_O_2_ or oxygen, were developed recently, also in our laboratory. On the other hand, F-TEDA-BF_4_ possesses unlimited potential as a catalyst or reagent in various condensations and coupling reactions. Up to now reported discoveries illustrate the really amazing possibilities of the organic molecule skeleton building reactions mediated by F-TEDA-BF_4_. It seems that many research groups have already recognized this fact, since a considerable number of recent papers reviewed in the present account are dedicated to this matter. 
